# Selection of Flax Genotypes for Pan-Genomic Studies by Sequencing Tagmentation-Based Transcriptome Libraries

**DOI:** 10.3390/plants12213725

**Published:** 2023-10-30

**Authors:** Elena N. Pushkova, Elena V. Borkhert, Roman O. Novakovskiy, Ekaterina M. Dvorianinova, Tatiana A. Rozhmina, Alexander A. Zhuchenko, Daiana A. Zhernova, Anastasia A. Turba, Arthur G. Yablokov, Elizaveta A. Sigova, George S. Krasnov, Nadezhda L. Bolsheva, Nataliya V. Melnikova, Alexey A. Dmitriev

**Affiliations:** 1 Engelhardt Institute of Molecular Biology, Russian Academy of Sciences, 119991 Moscow, Russia; pushkova18@gmail.com (E.N.P.); sashai@inbox.ru (E.V.B.); 0legovich46@mail.ru (R.O.N.); dvorianinova.em@phystech.edu (E.M.D.); zhernova.d@ya.ru (D.A.Z.); anastas.turba@gmail.com (A.A.T.); melarsoprol@mail.ru (A.G.Y.); sigova.ea@phystech.edu (E.A.S.); gskrasnov@mail.ru (G.S.K.); nlbolsheva@mail.ru (N.L.B.); 2 Moscow Institute of Physics and Technology, 141701 Moscow, Russia; 3 Federal Research Center for Bast Fiber Crops, 172002 Torzhok, Russia; tatyana_rozhmina@mail.ru (T.A.R.); ecovilar@mail.ru (A.A.Z.); 4 All-Russian Horticultural Institute for Breeding, Agrotechnology and Nursery, 115598 Moscow, Russia; 5 Faculty of Biology, Lomonosov Moscow State University, 119234 Moscow, Russia

**Keywords:** *Linum usitatissimum*, flax, pan-genome, Tn5, tagmentation-based libraries, transcriptome sequencing, genetic diversity

## Abstract

Flax (*Linum usitatissimum* L.) products are used in the food, pharmaceutical, textile, polymer, medical, and other industries. The creation of a pan-genome will be an important advance in flax research and breeding. The selection of flax genotypes that sufficiently cover the species diversity is a crucial step for the pan-genomic study. For this purpose, we have adapted a method based on Illumina sequencing of transcriptome libraries prepared using the Tn5 transposase (tagmentase). This approach reduces the cost of sample preparation compared to commercial kits and allows the generation of a large number of cDNA libraries in a short time. RNA-seq data were obtained for 192 flax plants (3–6 individual plants from 44 flax accessions of different morphology and geographical origin). Evaluation of the genetic relationship between flax plants based on the sequencing data revealed incorrect species identification for five accessions. Therefore, these accessions were excluded from the sample set for the pan-genomic study. For the remaining samples, typical genotypes were selected to provide the most comprehensive genetic diversity of flax for pan-genome construction. Thus, high-throughput sequencing of tagmentation-based transcriptome libraries showed high efficiency in assessing the genetic relationship of flax samples and allowed us to select genotypes for the flax pan-genomic analysis.

## 1. Introduction

In recent years, flax has gained increased attention due to the growing popularity of healthy lifestyles and environmentally friendly products. Flax seed is one of the richest plant sources of lignans and unsaturated fatty acids, which reduce the risk of cardiovascular diseases, cancers, and a number of other diseases [1,2,3,4,5,6]. Flax fiber and oil are used in the food, pharmaceutical, textile, polymer, composite, and medical industries, and the area of application of flax products is only expanding [6,7,8,9]. Efficient development of flax varieties with the desired traits requires knowledge of the plant’s genetics. High-quality genome assemblies are the basis for genetic research and molecular breeding of flax. The genome of the flax variety CDC Bethune was sequenced on the Illumina platform in 2012; the assembly size was 302 Mb with an expected genome size of 370 Mb [10]. The upgrade of the CDC Bethune genome assembly to the chromosome level was performed in 2018 using optical mapping and genetic maps [11]. Later, more complete and rather extended genome assemblies of other flax varieties were obtained using nanopore sequencing [12,13,14]. In addition, hundreds of flax varieties were sequenced using the Illumina and BGI platforms with short reads [15,16].

The assembly of near-complete genomes and the construction of pan-genomes are promising perspectives in plant genome sequencing [6,17,18]. Therefore, the next important step in flax genome research is to move towards a pan-genome constructed from high-quality genomes of a representative set of flax accession. The assembly of high-quality genomes usually requires the use of third-generation sequencing (Oxford Nanopore Technologies or Pacific Biosciences platforms) as well as significant computing power, so the cost of pan-genomic research can be quite high [19,20,21]. In this regard, it is necessary to select the right genetic material, which is the basis for the success of further work. It is important to form a sample set that most fully encompasses the diversity of flax based on morphological characteristics, geographical origin, and genetic relationship.

Cultivated flax is usually classified according to morphological characteristics and type of use as fiber, intermediate, crown, large-seeded, dehiscent (*Linum usitatissimum* convar. *crepitans*), and winter flax [22]. Cultivated flax is thought to have originated from the domestication of the wild flax, *Linum angustifolium* [23,24,25,26,27,28]. *L. angustifolium* produces fertile hybrids with *L. usitatissimum* and these species are genetically close [29]. Some researchers consider *L. angustifolium* not as a species but as a subspecies of *L. usitatissimum*–*L. usitatissimum* subsp. *angustifolium* (Huds.) Thell. [30,31]. In many other sources, such as the Flora of Eastern Europe [32] and Flora Europaea [33], web-resources The WFO Plant List (https://wfoplantlist.org/plant-list/, accessed on 14 September 2023), Plantarium (https://www.plantarium.ru/lang/en.html, accessed on 14 September 2023), and INaturalist (https://www.inaturalist.org/, accessed on 14 September 2023), *L. angustifolium* is also not listed as a separate species, but refers to *Linum bienne*. However, the Russian school of botany considers *L. angustifolium* and *L. bienne* to be separate species [34]. Thus, morphologically, *L. usitatissimum* and closely related species are very diverse, and this diversity needs to be covered when creating a pan-genome.

Geographical origin is also important to consider when creating a plant sample set for a pan-genomic study. Sinskaya suggested the existence of three centers of origin for cultivated flax. Indian, Abyssinian, and Mediterranean crown flax were derived from the Indian center of origin. Light-seeded Khiva crown flax, Pamir dwarf crown flax, and Middle Volga and North Russian intermediate flax originated from the Indo-Afghan center. The Colchis center may have given rise to Caucasian crown flax, steppe Ukrainian and Anatolian intermediate flax, and Western European fiber flax [35].

Based on morphology and geographical origin, a primary set of flax samples can be generated for pan-genomic studies. However, in genetic banks, from which seeds are often obtained for research, there are sometimes errors in the species identification of accessions, which can lead to incorrect results. For flax, a notable example of such an error is the accession LIN 1655 from the collection of the Leibniz Institute of Plant Genetics and Crop Plant Research (IPK, Gatersleben, Germany), which has been used in several phylogenetic studies of the genus *Linum*. LIN 1655 was labeled as *Linum stelleroides* (section *Stellerolinum* Juz. ex Prob.) but belongs to the *Linum perenne* group (section *Adenolinum* (Reichenb.) Juz.) [36]. In addition, there is a problem of accession heterogeneity, which is particularly acute for landraces and wild flax. In this respect, it is necessary to carry out a genetic analysis of the primary sample set to select genotypes that best cover the genetic diversity and are typical representatives of the studied flax accessions. It is advisable to perform such analysis on a few individual plants of each accession pre-selected for pan-genome construction. DNA sequencing-based approaches, including whole-genome, reduced-representation, and targeted sequencing, are effective for obtaining data on plant DNA polymorphisms and estimating the genetic relationship of accessions. There are also approaches for plant phylogenetic studies based on transcriptome sequencing, which allow the determination of sequences of genes expressed in specific tissues and the identification of polymorphisms in protein-coding genes [37]. In recent years, cost-effective library preparation methods for high-throughput sequencing have been developed, and a number of them are based on the use of the Tn5 transposase (tagmentase) [38,39]. In our work, a method based on the sequencing of cDNA libraries prepared using tagmentase [40] was adapted to select accessions and typical plants that sufficiently encompass flax diversity for subsequent pan-genome construction.

## 2. Results

### 2.1. Preparation of cDNA Libraries Using the Tn5 Transposase

**Figure 1:**
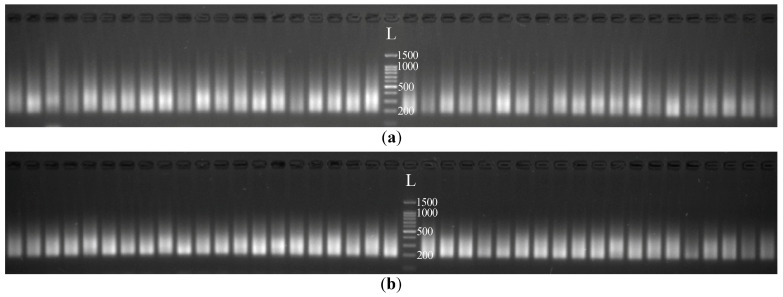
Length distribution of tagmentation-based cDNA libraries visualized by agarose gel electrophoresis: (**a**) cDNA libraries prepared using the KAPA HiFi HS (Roche) polymerase; (**b**) cDNA libraries prepared using the Tersus (Evrogen) polymerase. L–100 + bp DNA Ladder (Evrogen).

Preparation of 288 cDNA libraries for 44 flax accessions (individual cDNA libraries for each of 3–6 plants of each accession, in total 192 plants) was performed. The Tn5 transposase was used for the fragmentation of RNA/DNA heteroduplexes and the ligation of synthetic oligonucleotides to the resulting fragments. Further, two variants of DNA polymerases (KAPA HiFi HS (Roche, Switzerland) or Tersus (Evrogen, Russia)) were used for the amplification of cDNA libraries. To evaluate the quality of the obtained cDNA libraries, we analyzed the length distribution of cDNA fragments by agarose gel electrophoresis (Figure 1). The majority of cDNA fragments were 200–500 bp in length for both KAPA HiFi HS (Roche) and Tersus (Evrogen) polymerases. The use of KAPA HiFi HS (Roche) allowed the amplification of fragments of quite long length—up to 1000 bp (Figure 1a). Such a length was not obtained using Tersus (Evrogen) (Figure 1b). However, as we performed size selection by isolating 250–500-bp fragments from the gel (optimal for the Illumina sequencing), differences in the ability of the polymerases to amplify long fragments did not play a significant role in our work.

### 2.2. Mapping the Obtained Illumina Reads to the Flax Genome

**Figure 2:**
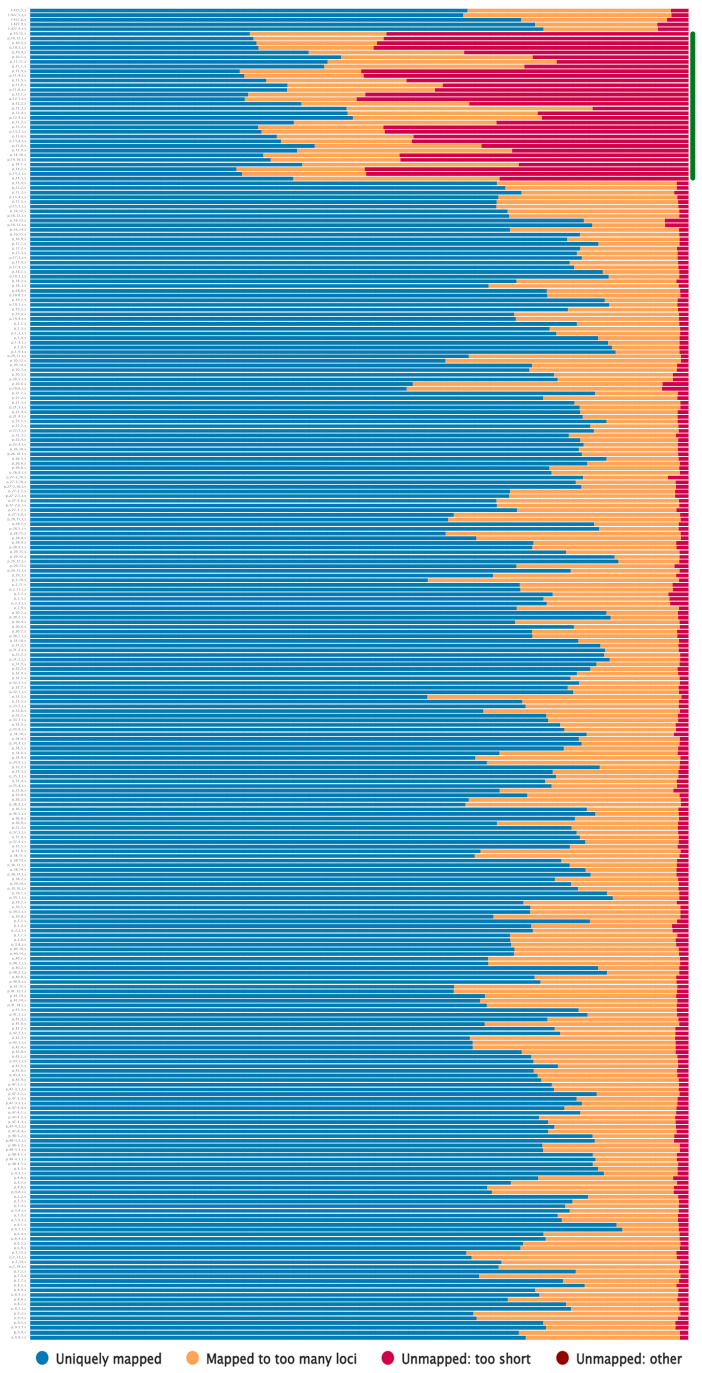
Results of mapping the obtained transcriptome reads to the *L. usitatissimum* genome (CDC Bethune, GCA_000224295.2). Samples for which fewer reads were mapped compared to the main sample set are marked by the green line to the right. “Unmapped: other” reads were almost absent.

Illumina sequencing resulted in an average of 1.5 M 75-bp reads for each of the 288 cDNA libraries. For most samples, on average, about 78% of the reads were uniquely mapped to the CDC Bethune genome assembly (GCA_000224295.2) (Figure 2). About 18% of the reads were mapped to many loci; they may represent multicopy genes, which are expected since the whole-genome duplication occurred during the origin of *L. usitatissimum* [10,27,41]. Exceptions were 32 samples (plants of accessions p_10, p_11, p_12, p_13, and p_14), for which on average only about 40% of the reads were uniquely mapped. According to the genbank data, these samples belonged to *L. angustifolium* with accession numbers K-6060, K-6065, K-6066, K-6077, and K-6085. However, plants from these accessions were phenotypically very different from the other accessions in the studied sample set (Supplementary Figure S1) and did not bloom in the first year of vegetation. For these accessions, about 50% of the reads did not map to the CDC Bethune genome, also indicating that they are significantly different from *L. usitatissimum* (for the rest of the sample set, the proportion of unmapped reads was 4% on average).

### 2.3. Gene Coverage Profiles

**Figure 3:**
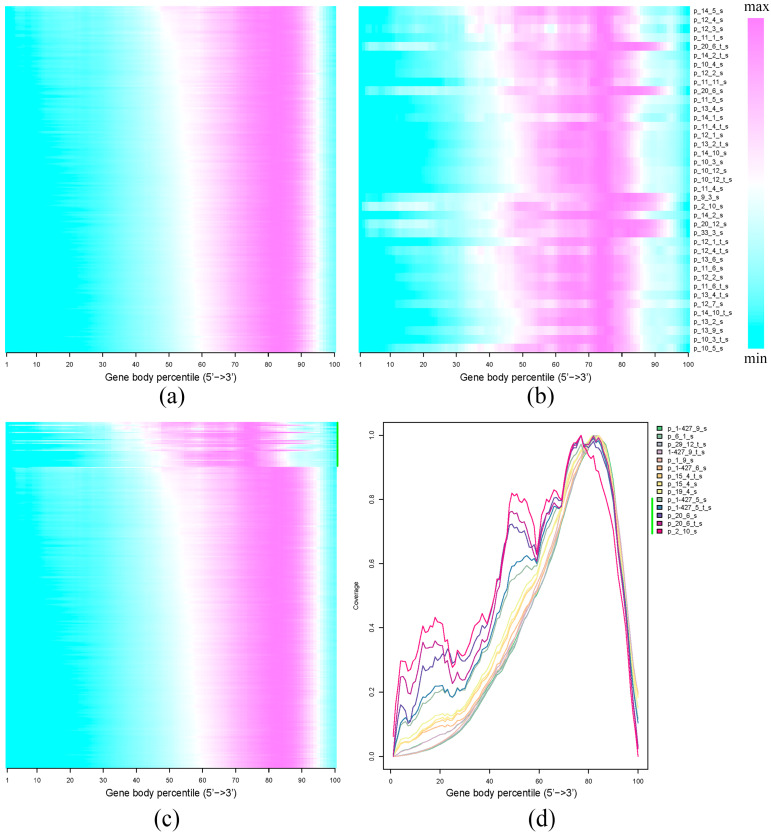
Gene coverage profiles for the reads obtained by transcriptome sequencing of flax samples. (**a**) Typical gene coverage profiles for the majority of samples examined; (**b**) samples for which gene coverage profiles differed from the main sample set; (**c**) typical gene coverage profiles for the majority of samples examined (lower part) versus profiles differed from the main sample set (upper part, shown by the green line on the right); (**d**) typical gene coverage profiles for the majority of samples examined versus profiles differed from the main sample set (shown by the green line to the left of sample names).

We evaluated the gene coverage profiles for the obtained transcriptome reads (Figure 3). For most flax samples (Figure 3a), the distribution of the reads was uneven, and the highest coverage appeared to be skewed towards the 3’-end of genes. This related to the specifics of library preparation—the reverse transcription variant with the dT30VN primer was used, and therefore the reaction started from the poly-A tail corresponding to the 3’-end. As a result, this region was best presented in the sequencing data. The limitation of this approach is that we can search for polymorphisms closer to the 3’-end of a gene rather than along the entire gene length, and thus obtain reliable data for fewer polymorphic sites. On the other hand, since the majority of the reads fell within approximately 30% of gene length (from the 65th to 95th gene body percentile from the 5’-end to the 3’-end, Figure 3a), we need fewer reads per sample to maintain sufficient coverage at the 3’-end to obtain reliable results. For some samples, we observed not a smooth decrease in gene coverage from the 3’-end to the 5’-end but a decrease with several local increases. Gene coverage data for such samples are shown in Figure 3b, and in Figure 3c,d these samples (marked by the green line to the left of sample names) are shown in comparison with samples with typical coverage distribution. The local increases in gene coverage were likely associated with the overrepresentation of a number of gene sequences due to the poor quality of the RNA.

### 2.4. Identification of DNA Polymorphisms and Clustering of Flax Samples

**Figure 4:**
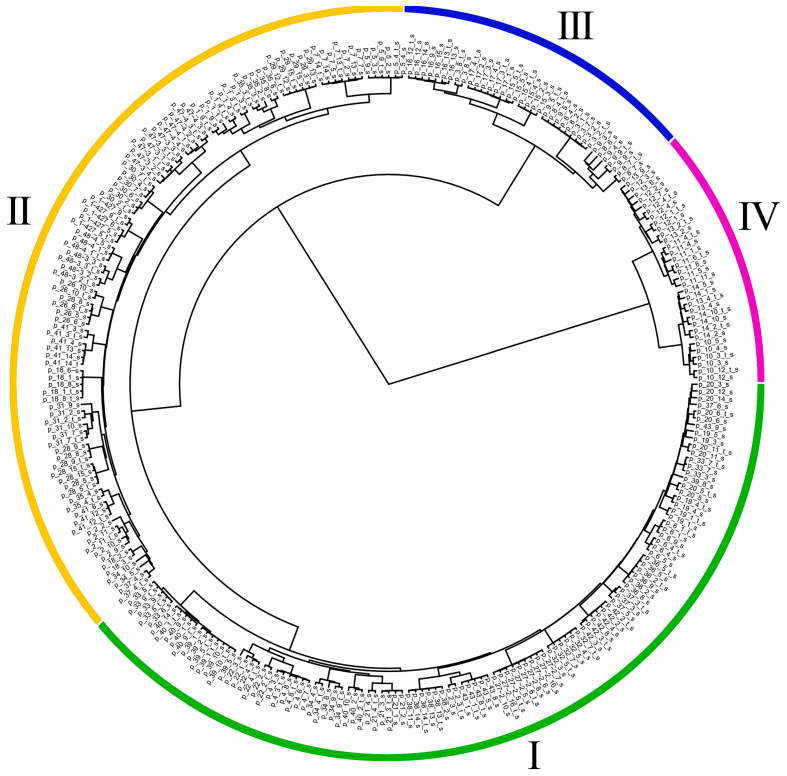
Dendrogram of plants of 44 flax accessions based on polymorphisms identified in transcriptome sequencing data. The number after the first underscore (_) corresponds to the accession number in Table 1, and the number after the second underscore corresponds to the plant number. Samples prepared using the Tersus (Evrogen) polymerase have the letter ‘t’ at the end of the name.

We searched for DNA polymorphisms in 288 sequenced flax transcriptomes. The number of identified polymorphisms with an average coverage of more than 10 reads was about 28 thousand (Supplementary Table S1). Thus, using transcriptome analysis with tagmentation-based library preparation, we obtained data on a large number of polymorphic DNA sites, which is important for the reliable assessment of the genetic relationship of the studied samples. Based on the obtained DNA polymorphism data, we estimated the genetic distances between the studied samples of 44 flax accessions and constructed a dendrogram (Figure 4, Supplementary Figure S2, Table 1). Four clusters were identified. Cluster I included both oilseed flax (intermediate and crown flax) and fiber flax accessions. Cluster II consisted mainly of oilseed flax (intermediate and crown flax) and also included the convar. *crepitans* accessions. Large-seeded flax accessions appeared in clusters I and II. Cluster III consisted of *L. angustifolium*/*L. bienne*. The most isolated cluster IV was formed by the group of accessions p_10, p_11, p_12, p_13, and p_14, which belong to *L. angustifolium* according to the genbank data. It is worth noting that *L. angustifolium*/*L. bienne* from cluster III (p_3, p_8, p_9, p_15, p_16, and p_17) were genetically closer to *L. usitatissimum* than to *L. angustifolium* from cluster IV. For further pan-genomic analysis, it is advisable to select flax genotypes that fully represent all clusters reflecting the diversity of *L. usitatissimum*/*L. angustifolium* and the dendrogram we obtained contained the necessary information.

Sequenced cDNA libraries prepared from the same RNA but using different DNA polymerases (KAPA HiFi HS (Roche) or Tersus (Evrogen)) were usually located close to each other on the dendrogram. This indicates the reproducibility of the results obtained using different polymerases. It is worth noting that most samples with atypical gene coverage profiles clustered with samples of the same accession with typical coverage profiles. This also indicates the robustness of the performance of the applied Tn5-based library preparation.

In most cases, plants of the same accession clustered together and formed small subclusters. This pattern was observed for accessions p_1, p_4, p_5, p_6, p_7, p_10, p_11, p_14, p_16, p_17, p_21, p_26, p_28, p_29, p_30, p_31, p_32, p_36, p_38, p_42, p_47, p_48, and p_1-427. However, for a part of the accessions, plants were not clustered together but interspersed in a subcluster with plants of other accessions (p_8, p_9, p_12, p_19, and p_20) or appeared in different subclusters of the same cluster (p_3, p_13, p_15, p_18, p_22, p_33, p_35, p_39, p_40, p_41, and p_43) or different clusters (p_2, p_34, and p_37). For such accessions, our study was one of the key steps in the selection of typical representatives for their further use in pan-genomic analysis.

### 2.5. Species Identification for Some Accessions

Phenotypically, *L. angustifolium* plants from cluster IV (Supplementary Figure S1a) differed significantly from *L. angustifolium*/*L. bienne* plants from cluster III and *L. usitatissimum* plants from clusters I and II (Supplementary Figure S1b). To verify the species identity of the *L. angustifolium* plants from cluster IV (accessions p_10, p_11, p_12, p_13, and p_14), we mapped the transcriptome sequencing data to the reference internal transcribed spacer (ITS) sequence of *L. usitatissimum*. Notably, no reads were mapped to a part of the *L. usitatissimum* ITS sequence. We determined the consensus sequences from the reads mapped to the reference ITS region for p_10, p_11, p_12, p_13, and p_14. Their BLAST analysis showed that these sequences were close to *L. perenne* and related species (99–100% identity) but not to *L. usitatissimum* and *L. angustifolium*. The transcriptome sequencing data obtained for p_10, p_11, p_12, p_13, and p_14 were then mapped to the reference ITS sequence of *L. perenne*. In this case, the reads were mapped to the whole ITS, and we determined complete ITS sequences for the studied accessions of cluster IV. Their BLAST analysis confirmed that these sequences are similar to those of the ITS of *L. perenne* and closely related species but not to the ITS sequences of *L. usitatissimum* and *L. angustifolium*. The same analysis for the *L. angustifolium*/*L. bienne* samples from cluster III showed that these samples are closer to *L. usitatissimum* and *L. angustifolium* but not to other flax species.

More than a year after the p_10 seeds were planted, several plants of this accession entered the flowering phase (Supplementary Figure S3). The plants were very different from the description of *L. angustifolium* in the Flora of the USSR, vol. 14 [34] and Flora Europaea, vol. 2 [33]. Most notably, the p_10 plants were heterostylous, the stigmas were capitate, and the sepals were acute or obtuse (often mucronate), in contrast to the narrowly acute sepals of *L. angustifolium*/*L. bienne*. Characteristic of p_10 were the obtuse outer and rounded mucronate sepals, 3.5–5 mm long, about 4 times shorter than the petals. The leaves were up to 5 cm long, extending from the stem. The indicated characters led to *L. perenne* L., according to the key in the Flora of the USSR, vol. 14 [34]. According to the key of Flora Europaea, vol. 2 [33], p_10 was also a member of the *L. perenne* group. The main difference between *L. perenne* and *L. angustifolium*/*L. bienne* is the capitate stigmas and the heterostylous flowers. Thus, the morphological analysis also showed that one of the members of cluster IV is not *L. angustifolium* (section *Linum*) but a member of the *L. perenne* group (section *Adenolinum*).

Based on the genetic and morphological data obtained, we concluded that the accessions of cluster IV (p_10, p_11, p_12, p_13, and p_14) do not belong to *L. angustifolium,* and it is inappropriate to include them in the pan-genomic analysis of *L. usitatissimum* and closely related species.

Thus, the transcriptome sequencing data of tagmentation-based cDNA libraries allowed us to obtain data on a significant number of DNA polymorphisms in the expressed genes, assess the genetic relationship for the studied flax plants, and select the most representative genotypes for further pan-genomic analysis.

## 3. Discussion

The aim of our study was to assess the genetic relationship between accessions of the primary flax sample set for pan-genomic analysis and select the most representative genotype of each accession. For the selected genotypes, accurate and contiguous genomes should then be obtained for the flax pan-genome construction. An approach based on the use of Tn5 was applied to prepare cDNA libraries from flax leaves. Tn5 is a transposase that allows the simultaneous fragmentation of double-stranded DNA or RNA/DNA heteroduplexes and the addition of oligonucleotides necessary for the amplification and sequencing of the library [40,42]. After PCR with primers containing barcodes and regions required for sequencing, the libraries were ready to use. The tagmentation step is present in a number of commercial reagent kits for the preparation of DNA libraries for high-throughput sequencing (including Illumina, NEB, and Qiagen), and the advantage of this approach is that the sample preparation is quick and easy. To reduce the cost of commercial reagent kits, methods with in-house reagents were developed. For example, Tn5-based techniques to prepare DNA libraries for high-throughput sequencing were proposed [43,44,45]. In addition, Tn5 was also applied to transcriptome libraries [38,39,46,47]. It was shown that Tn5 can tagmentate not only DNA/DNA duplexes but also DNA/RNA heteroduplexes [40,48,49]. The advantage of this approach is that there is no need to pre-amplify the DNA/RNA heteroduplex to obtain a DNA/DNA duplex (pre-amplification is an additional step that can lead to a shift in transcript representation compared to the original sample). Therefore, we used DNA/RNA tagmentation to generate cDNA libraries to select the most representative plants for the flax pan-genome construction.

The application of the chosen approach allowed us to obtain data on the polymorphisms of genes expressed in flax leaves and cluster the plants of the studied sample set based on these results. For the accessions of the most isolated cluster IV (Figure 4), a misidentification of species affiliation was shown. The accessions of this cluster belonged not to *L. angustifolium* but to *L. perenne*. As a result, we were able to exclude these accessions from the sample set for further pan-genomic analysis of *L. usitatissimum* and closely related species. The accessions that truly belong to *L. angustifolium* formed cluster III, which was separated from clusters I and II of *L. usitatissimum* representatives. This result is quite reasonable. Two clusters (I and II) were identified for cultivated flax, one of which included fiber flax together with oilseed flax, while the other cluster did not contain any fiber flax.

The result obtained is in line with Sinskaya’s ideas about the separation of a group that includes fiber, intermediate, and crown flax, as well as groups that contain intermediate and crown flax, but not fiber flax [35]. The clustering of flax samples from the present study was also in general agreement with the estimates of relationships obtained for flax based on molecular genetic methods. Most works distinguish a group of fiber flax, which also includes flax of other morphological forms, and a group/groups of intermediate and crown flax, in which fiber flax is practically absent. The study of 407 cultivated flax samples from all over the world using microsatellite markers showed that the genotypes could be divided into three main groups: The first contained predominantly oilseed flax and was subdivided into three subgroups (South Asian, Western European, and South American flax); the second consisted of North American and European flax, also predominantly of the oilseed type; the third included North American and Eastern European accessions, most of which were fiber flax [50]. Analysis of polymorphisms of 424 genes involved in the synthesis of cell wall components, lignans, and fatty acids and encoding transporters for 191 flax varieties also revealed the division of samples into linseed and fiber flax groups [51]. The study of 306 flax accessions of different geographical origins and morphotypes using genome sequencing showed the separation of oilseed and fiber types of flax and suggested that kryazhs (Russian heritage landraces) originated from both the Indo-Afghan center and Fertile Crescent [15]. Whole-genome sequencing of 200 flax cultivars with high phenotypic polymorphism and different geographical origins revealed that oil flax is the ancestor of cultivated flax, and the oil-fiber group is intermediate between oil and fiber flax [16]. It can be concluded that the division of flax into groups obtained in the present work is consistent with the results of other authors and that the method of sample preparation we used allowed us to obtain adequate data on the relationships between flax genotypes.

For phylogenetic studies in plants, whole-genome sequencing is a highly informative approach [52]. It provides data on most of the DNA polymorphisms of a given genotype, but the cost per sample is significant. Our task was to examine 3–6 individual plants for each of the 44 flax accessions, and the price of whole-genome analysis was too high for such a large sample set.

Sequencing of organelle DNA, as well as ribosomal DNA, is commonly used to assess the genetic relationship of samples. However, such sequences are often insufficiently polymorphic to study groups of closely related plants, including flax [27,53]. This approach would allow us to detect an error in species identification for the accessions of cluster IV (*L. perenne* instead of *L. angustifolium*). However, it would not allow an adequate comparison between plants of the same accession and between plants of closely related accessions. Therefore, this method was not used.

Another commonly used technique to assess the genetic relationship of samples is reduced-representation sequencing. It can significantly reduce the cost per sample compared to whole-genome sequencing by examining only a portion of a genome [52,54]. However, this approach often requires the selection of optimal restriction enzymes and the optimization of sample preparation methods for a particular plant species/genus [37]. In addition, it provides data on polymorphisms that are predominantly localized in non-coding regions of a genome rather than in gene exons, which may also be less informative than the study of polymorphisms in coding regions. At the same time, reduced-representation sequencing was successfully applied to flax for SNP identification and DNA marker development [55,56].

Sequencing of gene sets with a sufficient number of polymorphic sites is a fairly effective approach for characterizing sample set diversity, but the development of such gene sets requires significant bioinformatics analysis and optimization of the methods for isolation of the required sequences from a genome [37]. For members of the genus *Linum*, the evaluation of such a gene set showed good results [57]. However, it is unclear how effective the set would be for closely related samples.

Sequencing of plant transcriptomes provides data on the polymorphism of protein-coding genes, which can be used in phylogenetic studies and for the assessment of the genetic relationship of plants [58,59,60,61,62]. This allows the comparative analysis of both genetically close samples of the same species and representatives of different species of the same genus and does not require preliminary bioinformatics work for previously unexplored plant species. The disadvantages of this approach are the difficulties associated with working with RNA: more complex isolation compared to DNA, the need to store plant material and RNA at low temperatures, and the need to collect the same plant material at the same developmental stage [37]. To evaluate a flax sample set for pan-genomic analysis, we chose a technique based on transcriptome sequencing with a preparation option using the in-house Tn5 transposase. This approach reduces the cost of sample preparation compared to commercial kits and allows the generation of a large number of cDNA libraries in a short time. The method we used showed high efficiency in assessing the genetic relationships of both closely related and genetically distant flax plants. For half of our accessions, the analysis led to the exclusion of plants that were not typical representatives of the examined accessions. As a result, our study allowed us to select flax plants whose progeny will be sequenced to create a pan-genome of *L. usitatissimum* and closely related species.

Thus, the technique used in our work allowed us to assess the genetic relationship of the flax accessions and to select plants that were typical of these accessions. It is an alternative to whole-genome and reduced-representation sequencing. This method can be applied to different flax plant tissues (including stems and seeds, which are used in various industries and may be of particular interest for research). This approach can also be effectively transferred to other plant species to analyze the polymorphism of genes expressed in organs and tissues of interest.

## 4. Materials and Methods

### 4.1. Plant Material

For the pan-genomic analysis, we selected a primary sample set of 44 flax accessions to cover the diversity of *L. usitatissimum* and closely related species, taking into account morphological traits, geographical origin, and genetic data [22,23,24,25,26,27,28]. The sample set included different forms of flax (fiber, intermediate, crown, large-seeded, dehiscent (convar. *crepitans*), and winter flax) representing ancient regions of flax cultivation, as well as a number of modern, promising varieties (Table 1).

Flax plants of the selected accessions were grown in 15-liter pots under 16 h of light per day. Leaves from the middle part of the shoot were collected from 4-week-old plants. Plant material was immediately frozen in liquid nitrogen and stored at −70 °C until RNA isolation. A total of 660 plant samples were collected (from 15 individual plants of each of the 44 flax accessions).

### 4.2. Preparation and Sequencing of cDNA Libraries

Leaves were ground using a TissueLyser II homogenizer (Qiagen, Chatsworth, CA, USA) in lysis buffer from the ExtractRNA Kit (Evrogen) and then RNA was isolated using this kit according to the manufacturer’s protocol with DNAase I treatment (Qiagen). RNA was then purified using the CleanRNA Standard Kit (Evrogen). RNA quality was verified by agarose gel electrophoresis and on a 2100 Bioanalyzer (Agilent Technologies, Santa Clara, CA, USA). RNA concentration was estimated using a Qubit fluorometer (Thermo Fisher Scientific, Waltham, MA, USA).

For each of the 44 flax accessions, 3–6 cDNA libraries were prepared for the most phenotypically typical plants that produced seeds (seeds are required to obtain plant material for pan-genomic analysis). Preparation of cDNA libraries was performed according to the protocol by Di et al. [40] with some modifications, which were mainly related to the use of reagents from other manufacturers. The reverse transcription reaction was performed according to the following protocol: 2 µL of 10 µM dT30VN [40] was added to 5 µL of RNA with a concentration of 20–30 ng/µL, incubated for 2 min at 70 °C, and placed on ice; then a reaction mixture consisting of 1× First strand buffer (Evrogen), 1 mM dNTPs (Evrogen), 2 mM DTT (Evrogen), 10 U RNase inhibitor (Syntol, Russia), 200 U Mint reverse transcriptase (Evrogen), and 1 µM TSO (5′-AAGCAGTGGTATCAACGCAGAGAGTACATrGrG+G-3′) [41] was added, incubated for 1 h 30 min at 42 °C and 15 min at 70 °C, and immediately transferred to ice.

The preparation of cDNA libraries was based on the use of the in-house Tn5 transposase with preannealed oligonucleotides obtained according to Picelli et al. [42]. This allows the fragmentation of RNA/DNA heteroduplexes [40] and the addition of oligonucleotides necessary for further preparation of cDNA libraries and sequencing. To assemble oligonucleotides with the Tn5 transposase, the following mixture was used: 0.125 volume of a 100 mM equimolar Tn5-A (5′-TCGTCGGGGCAGCGTCAGATGTGTGTATAAGAGACAG-3′ preannealed with 5′-TCTACACACATATTCTCTGTC-3′) and Tn5-B (5′-GTCTCTCGTGGGCGCTCGGAGATGTGTGTATAAGAGACAG-3′ preannealed with 5′-TCTACACATATATTCTTCTGTC-3′) oligonucleotides in TE, 0.4 volume of 100% glycerol, 0.12 volume of Tn5 dialysis buffer, and 0.36 volume of 1.85 mg/mL Tn5. The mixture was incubated at room temperature for 60 min, and the assembled Tn5 was stored at −20 °C before use. Tagmentation reaction was performed in 10 µL of reaction mixture containing 2 µL of 5× TAPS buffer (50 mM TAPS-NaOH), 25 mM MgCl_2_, 50% *v*/*v* DMF (pH 8.5), 0.125 µL Tn5, 0.85 mM ATP, and 6 µL of RNA/DNA heteroduplexes obtained after reverse transcription. The mixture was prepared on ice. It was further incubated for 7 min at 55 °C and immediately transferred to ice. Then, 5 µL of 0.2 mM SDS was added to the sample, incubated for 7 min at 55 °C, and immediately transferred to ice. The reaction products were diluted twofold. The gap-filled reaction was carried out in 10 µL of reaction mixture containing 1× First strand buffer (Evrogen), 0.3 mM dNTP (Evrogen), 100 U of Mint reverse transcriptase (Evrogen), and 8 µL of the Tn5 transposase reaction product. The mixture was then incubated for 15 min at 42 °C and 15 min at 70 °C and immediately transferred to ice. The reaction products were diluted twofold. PCR was performed using Nextera XT v2 primers: AATGATACGGCGACCACCGAGATCTACACACAC[i5]TCGTCGGCAGCAGCGTC and CAAGCAGAAGAGACGGCATACGAGAT[i7]GTCTCTCGTGGGCTCGG, where i5 and i7 are barcode sequences. PCR was performed with two different polymerases in 20 µL of reaction mixture containing 0.4 units/µL of the KAPA HiFi HS polymerase (Roche) or 1× Tersus polymerase (Evrogen), 1× KAPA polymerase buffer (Roche) or 1× Tersus polymerase buffer (Evrogen), 0.4 µM of each of i5 and i7 primers (Evrogen), 0.3 µM dNTP (Roche or Evrogen), and 5 µL of the product after the reaction with Tn5 and gap-filled reaction. The following amplification program was used on the MiniAmp Thermal Cycler amplifier (Thermo Fisher Scientific): 5 min—72 °C; 2 min 45 s—98 °C; 20 cycles: 15 s—98 °C, 30 s—62 °C, and 1 min 30 s—72 °C. A total of 192 cDNA libraries were prepared using the KAPA HiFi HS polymerase (Roche). For half of the same RNA samples, 96 cDNA libraries were prepared using the Tersus polymerase (Evrogen). The quality of the cDNA libraries was assessed by electrophoresis in a 2% agarose gel, and the concentration was measured on a Qubit fluorometer (Thermo Fisher Scientific). The cDNA libraries were mixed equimolarly, and fragments of 250–500 bp in length were isolated from the gel, followed by cDNA purification using the GeneJET Gel Extraction Kit (Thermo Fisher Scientific) according to the manufacturer’s protocol. The resulting total pool of cDNA libraries was purified twice on AMPure XP beads (Beckman Coulter, Brea, CA, USA) in a 1:1.5 (sample:beads) ratio. The concentration and quality of the prepared pool of cDNA libraries were assessed using a Qubit fluorometer (Thermo Fisher Scientific) and a 2100 Bioanalyzer (Agilent Technologies). The pool was diluted to a concentration of 4 nM and sequenced on NextSeq 500 (Illumina, San Diego, CA, USA) with 75 cycles of sequencing.

### 4.3. Bioinformatic Processing of Data

The RNA-seq Illumina reads were trimmed and filtered using Trimmomatic 0.39 [63] (min. 3′-quality 28, a sliding window of 4 bp with an average quality of at least 17, min. trimmed read length of 40 bp) and then mapped to the flax genome of variety CDC Bethune (NCBI database, assembly ASM22429v2/GCA_000224295.2) using the STAR2.7.2b mapper [64] in two-pass mode. Based on the results of the first pass, information on all splice junctions for all samples was collected, and a single catalog of splice junctions was formed, which was used in the second STAR pass. The mapping statistics were tabulated using MultiQC [65]. BAM files were sorted with samtools and then processed using the SplitNCigarReads tool (GATK 4.2.4.0) to split reads spanning introns. We then performed SNP calling using the freeBayes 1.3.2 tool [66] with a minimum variant allele frequency (VAF) value of 0.2, mapping quality and base quality thresholds of 20, and minimum coverage of 5×. Since the number of polymorphisms found was extremely large, the obtained VCF files were filtered by the PhredQ > 200 threshold to extract the most reliable variants. Next, for each sample, a vector with VAFs for all polymorphisms found was obtained, Euclidean distances between these vectors (i.e., samples) were calculated, and clustering of samples was performed using the Ward.D2 algorithm. The analysis was carried out in the R environment. The dendrogram visualization was prepared with the ggtree 3.6.2 package. The cut-off threshold for the separation of main clusters was chosen based on genetic distances and bootstrap values (Supplementary Figure S2). The bootstrap analysis was performed with pvclust [67].

ITS (internal transcribed spacer) sequences were used to identify the species affiliation of the flax accessions studied. The CLC Genomics Workbench tool (Qiagen) with default parameters was applied to map the reads obtained by transcriptome sequencing to the GenBank MK066769.1 (*L. usitatissimum* internal transcribed spacer 1, partial sequence; 5.8S ribosomal RNA gene, complete sequence; and internal transcribed spacer 2, partial sequence) and MK066736.1 (*L. perenne* internal transcribed spacer 1, partial sequence; 5.8S ribosomal RNA gene, complete sequence; and internal transcribed spacer 2, partial sequence) sequences. Blast analysis was performed for the consensus sequences obtained for the analyzed flax samples.

## Figures and Tables

**Table 1:** List of flax accessions of the primary sample set for pan-genomic analysis.

Number	Accession Name	Species/Form	Origin
p_1	K-1230	*L. crepitans*	Portugal
p_2	K-1531	*L. crepitans*	Spain
p_3	K-4731	*L. bienne*	-
p_4	Caesar	fiber	Russia
p_5	VNIIMK 620	intermediate	Russia
p_6	K-495	intermediate	Ukraine
p_7	K-1295	intermediate	Russia
p_8	K-5696	*L. angustifolium*	Iran
p_9	K-6036	*L. angustifolium*	Spain
p_10	K-6060	*L. angustifolium*	Italy
p_11	K-6065	*L. angustifolium*	France
p_12	K-6066	*L. angustifolium*	Canary Islands
p_13	K-6077	*L. angustifolium*	France
p_14	K-6085	*L. angustifolium*	Italy
p_15	K-6099	*L. angustifolium*	Tunisia
p_16	K-6375	*L. angustifolium*	Netherlands
p_17	K-6097	*L. angustifolium*	Portugal
p_18	Severny	intermediate	Russia
p_19	K-2864	fiber	Ukraine
p_20	K-470	fiber	Russia
p_21	Atlant	fiber	Russia
p_22	Alizee	fiber	Netherlands
p_26	K-1570	crown	China
p_27-2	K-2787	crown	Georgia
p_28	TR-42713	intermediate	Turkey
p_29	K-1119	crown	Armenia
p_30	K-432	crown	Kazakhstan
p_31	K-3774	crown	Uzbekistan
p_32	K-115	crown	Tajikistan
p_33	K-5543	large-seeded	India
p_34	K-568	crown	India
p_35	K-1024	large-seeded	Morocco
p_36	K-1146	crown	Ethiopia
p_37	K-519	large-seeded	Egypt
p_38	K-1743	crown	Egypt
p_39	Norlin	intermediate	Canada
p_40	K-550	crown	Argentina
p_41	K-1034	large-seeded	Argentina
p_42	K-1404	intermediate	Ukraine
p_43	K-3018	winter	Yugoslavia
p_47-3	47-3	crown	Russia
p_47-4	47-4	crown	Russia
p_48-3 and p_48-4	48-981	crown	Russia
p_1-427	1-427/10	crown	Russia

Note: The names of the accessions are given according to the names in the collection of the Institute for Flax (Torzhok, Russia).

## Data Availability

The raw sequencing data were deposited in the NCBI Sequence Read Archive (SRA) under the BioProject accession number PRJNA1018986.

## Supplementary Materials

The following supporting information can be downloaded at: https://www.mdpi.com/article/10.3390/plants12213725/s1, Figure S1: (a) Plants of the p_10 accession and (b) typical plants of *L. usitatissimum* and *L. angustifolium* (in different pots) from the analyzed flax sample set; Figure S2: Dendrogram of plants of 44 flax accessions based on polymorphisms identified in transcriptome sequencing data with approximately unbiased (au) *p*-values (left, red) and bootstrap probability (bp) values (right, green); Figure S3: Flowers of the p_10 accession (centimeter ruler is on the right); Table S1: DNA polymorphisms identified in the analyzed flax samples based on transcriptome sequencing data.
