# Supplementary material for: Selection of Flax Genotypes for Pan-Genomic Studies by Sequencing Tagmentation-Based Transcriptome Libraries
Source: Plants (Basel). 2023 Oct 30;12(21):3725. doi: 10.3390/plants12213725 (PMC10650069; doi:10.3390/plants12213725)
Supplement: Supplementary file 1 [file plants-12-03725-s001.zip › Figure S2 2023.10.18.pdf]

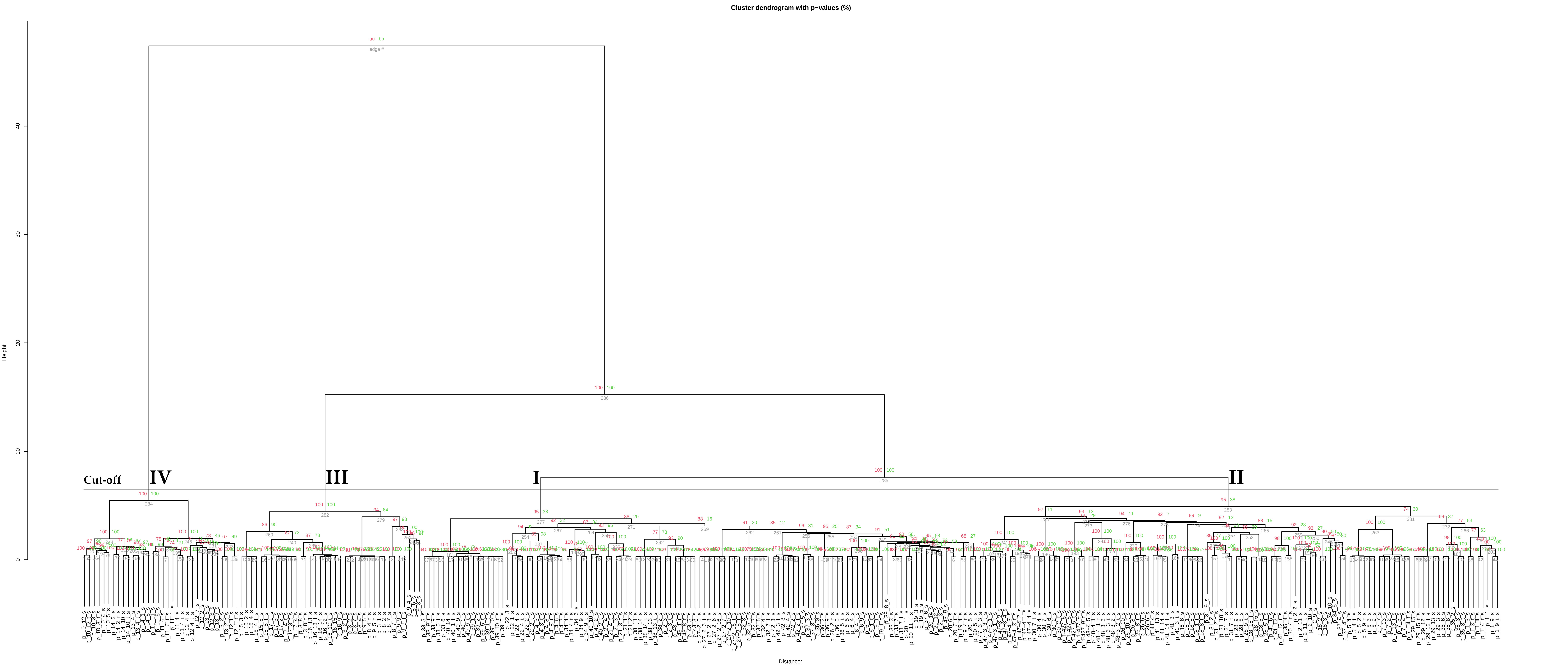


**Figure S2.** Dendrogram of plants of 44 flax accessions based on polymorphisms identified in transcriptome sequencing data. The number after the first underscore (_) corresponds to the accession number in Table 1, and the number after the second underscore corresponds to the plant number. Samples prepared using the Tersus (Evrogen) polymerase have the letter ‘t’ at the end of the name. Values at branches are approximately unbiased (au) p-values (left, red), bootstrap probability (bp) values (right, green), and cluster labels (bottom, gray). Roman numerals indicate the four clusters separated by the cut-off line.
