# Supplementary figures and images for: Selection of Flax Genotypes for Pan-Genomic Studies by Sequencing Tagmentation-Based Transcriptome Libraries
Source: Plants (Basel). 2023 Oct 30;12(21):3725. doi: 10.3390/plants12213725 (PMC10650069; doi:10.3390/plants12213725)

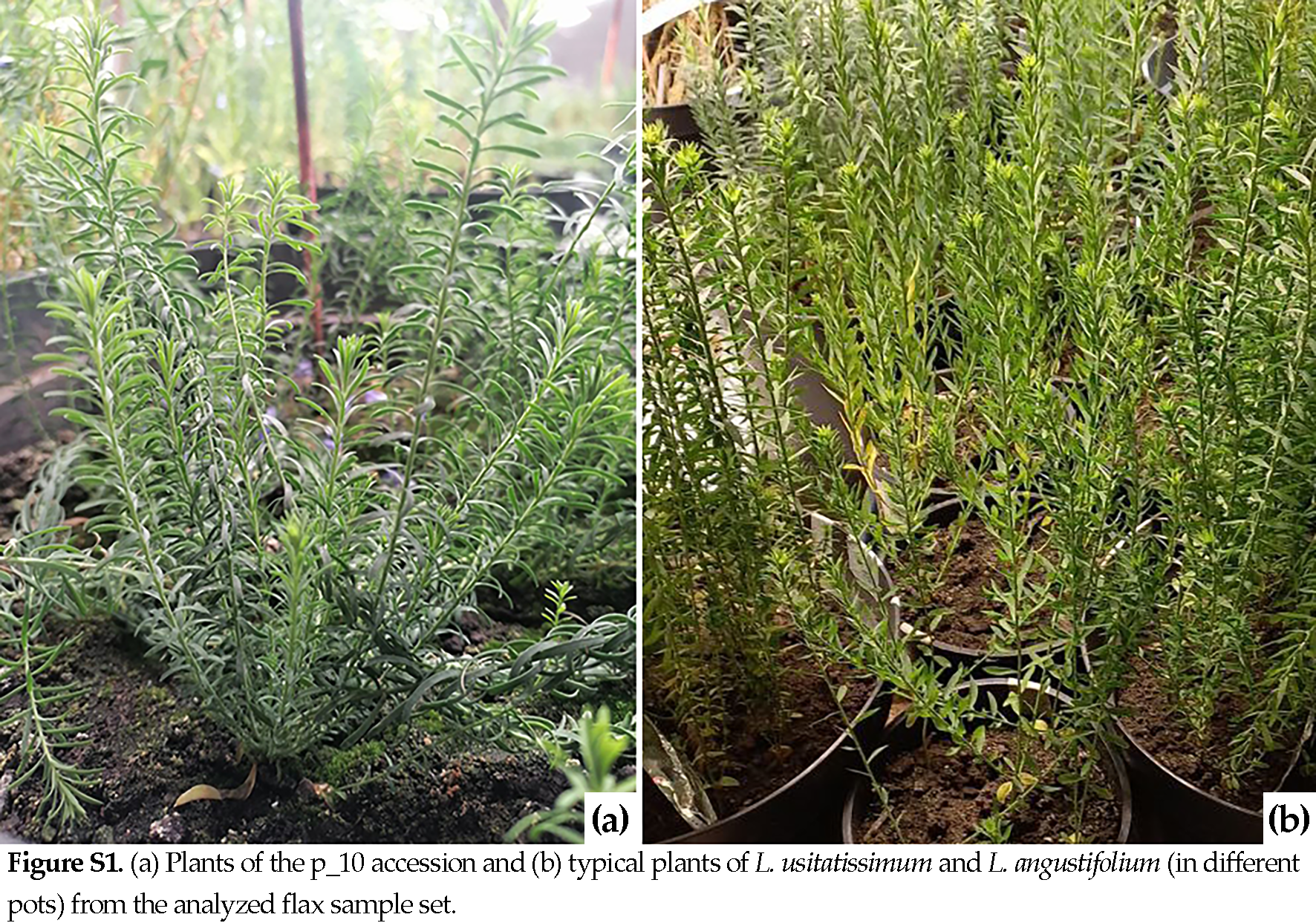

Supplement: Supplementary file 1 [file plants-12-03725-s001.zip › Figure S1 2023.10.17.tif]

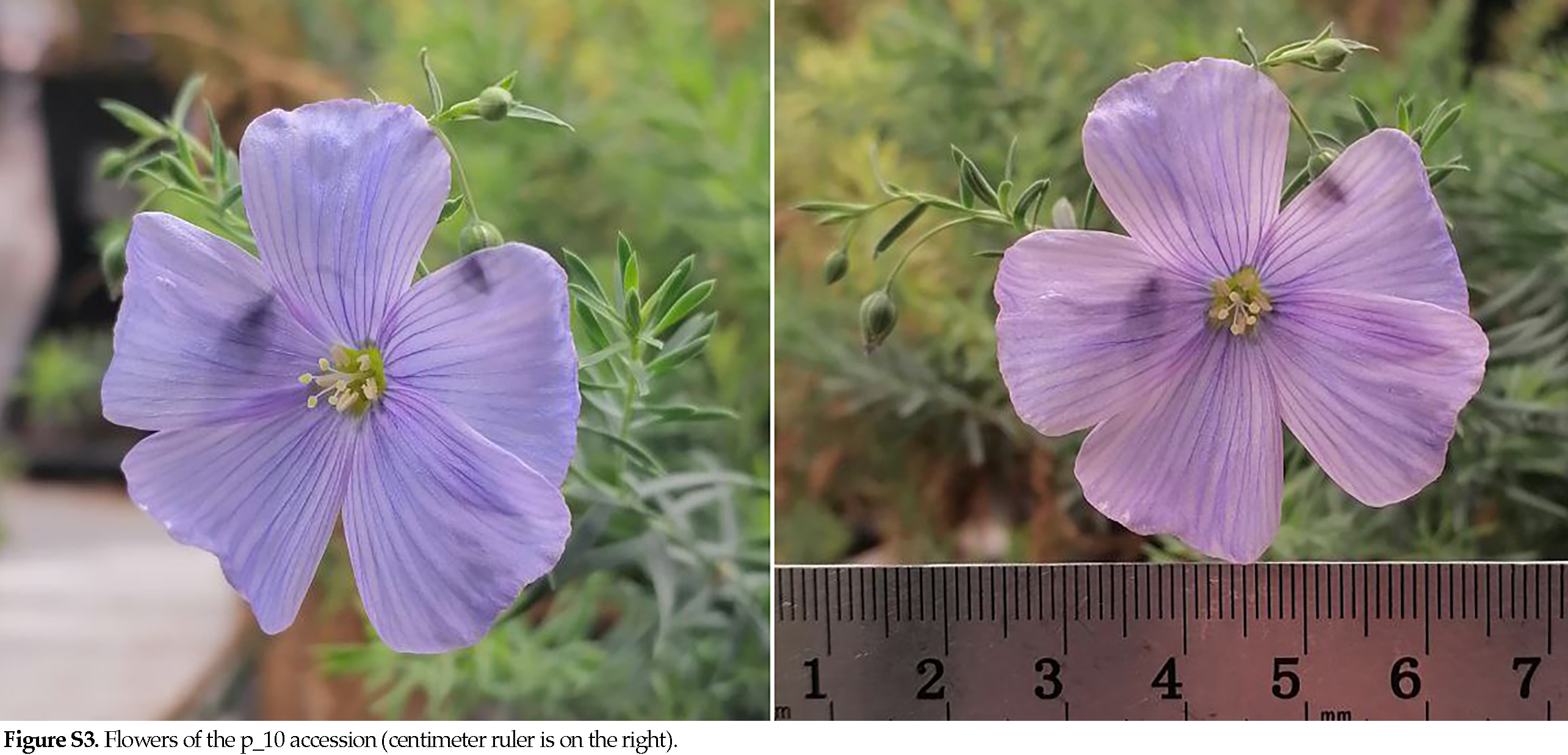

Supplement: Supplementary file 1 [file plants-12-03725-s001.zip › Figure S3 2023.10.17.tif]
